# Formation of Thiophene under Simulated Volcanic Hydrothermal Conditions on Earth—Implications for Early Life on Extraterrestrial Planets?

**DOI:** 10.3390/life11020149

**Published:** 2021-02-16

**Authors:** Thomas Geisberger, Jessica Sobotta, Wolfgang Eisenreich, Claudia Huber

**Affiliations:** Lehrstuhl für Biochemie, Department Chemie, Technische Universität München, Lichtenbergstraße 4, 85748 Garching, Germany; thomas.geisberger@tum.de (T.G.); Jessy.Sobotta@web.de (J.S.); wolfgang.eisenreich@mytum.de (W.E.)

**Keywords:** thiophene, acetylene, transition metal sulfides, hydrothermal conditions, early metabolism, origin-of-life

## Abstract

Thiophene was detected on Mars during the Curiosity mission in 2018. The compound was even suggested as a biomarker due to its possible origin from diagenesis or pyrolysis of biological material. In the laboratory, thiophene can be synthesized at 400 °C by reacting acetylene and hydrogen sulfide on alumina. We here show that thiophene and thiophene derivatives are also formed abiotically from acetylene and transition metal sulfides such as NiS, CoS and FeS under simulated volcanic, hydrothermal conditions on Early Earth. Exactly the same conditions were reported earlier to have yielded a plethora of organic molecules including fatty acids and other components of extant metabolism. It is therefore tempting to suggest that thiophenes from abiotic formation could indicate sites and conditions well-suited for the evolution of metabolism and potentially for the origin-of-life on extraterrestrial planets.

## 1. Introduction

Recent findings by the Curiosity mission have shown the existence of thiophene and some of its derivatives on Mars [1,2]. Within this context, different possibilities for their abiotic as well as biotic formation were discussed. Thiophene was even suggested as a biomarker in the search for life on Mars [2], whereas a hydrothermal abiotic origin was also considered [1]. Likewise, thiophenes are common pyrolysis products from meteoritic macromolecular materials. For example, these compounds are produced by aqueous and/or thermal alteration of carbonaceous chondrites like the Murchison meteorite [3,4]. On Earth, thiophenes can be detected in volcanic gas discharges and in fluid emissions related to hydrothermal systems [5]. In submarine basins, like the Guaymas basin, thiophene derivatives can be detected after hydrothermal pyrolysis of organic material [6,7]. Furthermore, thiophenes are suggested to be at least a part of the organic sulfur found in the globules of 2.72 Ga years old stromatolites of the Tumbiana Formation. In the Western Australian Dresser Formation, stromatolites are dated back to 3.5 Ga and are considered as one of the most ancient traces of life on Earth [8,9]. On an industrial scale, thiophene is produced from butane and sulfur at 560 °C, from sodium succinate and phosphorous trisulfide, and from acetylene and hydrogen sulfide at 400 °C on alumina [10].

We have shown earlier that acetylene is also an excellent source for primordial carbon fixation, especially in combination with carbon monoxide, e.g., for the synthesis of short chain fatty acids [11] and intermediates of extant carbon fixation cycles [12]. On Earth, acetylene is present in fumarolic exhalations [13]. It can also be found extra-terrestrially, for example, on Saturn’s moon Titan [14]. It was also proposed that explosive volcanism may have injected ∼6 × 10^12^ g/year of acetylene into the atmosphere of early Mars [15]. The importance of sulfides in an origin-of-life scenario is emphasized by Wächtershäuser’s Iron-Sulfur-World hypothesis [16]. Based on this hypothesis, we here report the facile abiotic formation of thiophene and some of its derivatives from acetylene and metallo-sulfides, especially NiS, under aqueous conditions at 105 °C. In context with the formation of potential building units and reaction networks for the emergence of metabolism under the same conditions [11,12] and capitalizing on recent hypotheses [2], the detection of extraterrestrial or terrestrial thiophenes could therefore indeed be indicative of early metabolic evolution under chemoautotrophic conditions.

## 2. Materials and Methods

All chemicals were purchased from Sigma Aldrich GmbH (Steinheim, Germany) in the highest purity available. Acetylene 2.6 (acetone free) was purchased from Linde AG (Pullach, Germany), and CO 2.5 and argon 4.6 were purchased from Westfalen AG (Münster, Germany). In a typical run (run 1, Table 1), a 125 mL glass serum bottle was charged with 1.0 mmol NiSO_4_·6H_2_O and closed with a silicon stopper. The bottle was evacuated three times and filled with argon, finally resulting in a de-aerated state. Subsequently, the bottle was filled with 3.5 mL argon-saturated water (calculated for a final volume of 5 mL) to dissolve the NiSO_4_ and with 1.0 mL argon-saturated 1 M Na_2_S solution. In this mixture, a precipitate of black NiS is immediately formed due to its low solubility constant of 1 × 10^−22^ [17,18] in aqueous solution. Furthermore, the bottle was filled with 0.5 mL 1 M NaOH solution, and finally with 120 mL of acetylene gas using gas-tight syringes for injection. The freshly precipitated NiS acted as a putative transition metal catalyst for the reaction and the molar variations of Na_2_S to NiSO_4_ resulted in free sulfide ions in the solution. In runs 10–12, 17, and 20, a mixture of 60 mL CO and 60 mL acetylene was used as gaseous phase. Instead of NiSO_4_·6H_2_O, runs 2, 11, and 14 were loaded with 1 mmol FeSO_4_·7H_2_O and runs 3, 12, and 15 were loaded with 1 mmol CoSO_4_·7H_2_O. In run 9, NiSO_4_·6H_2_O and FeSO_4_·7H_2_O were combined. Otherwise, the settings were identical to the above described procedure. Reactions were carried out at 105 °C. pH-Variations were achieved through the addition of 0.1–1.0 mL 1M H_2_SO_4_ or NaOH. For safety reasons (danger of explosion) and for technical reasons, the reactions were carried out at low pressure (1 bar) of acetylene. After 1 day (24 h) or 7 days, the reaction mixture was allowed to cool down and, after vigorous shaking, 1 mL was taken out and centrifuged at 10,000 rpm for 10 min. For the isolation of thiophenes, the supernatant and the solid residue were extracted separately with 1 mL ethyl acetate. The organic phases were dried over Na_2_SO_4_ and directly analyzed with gas chromatography-mass spectrometry (GC-MS). GC-MS analysis was performed with a GC-2010, coupled with MS-QP2010 Ultra (Shimadzu GmbH, Duisburg, Germany) with a 30 m × 0.25 mm × 0.25 µm fused silica capillary column (Equity TM5, Supelco, PA-Bellefonte, USA) and an AOC-20i auto injector. Temperature program and settings: 0–6 min at 40 °C; 6–25 min at 40–280 °C, 10 °C/min; injector temperature: 260 °C; detector temperature: 260 °C; column flow rate: 1 mL/min; scan interval: 0.5 sec; and injection volume 0.1 µL. For detection of thiophene derivatives, a larger injection volume of 3 µL was used. Peak assignment was achieved by comparison with the retention times and mass spectra of purchased reference compounds, as well as with data from the National Institute of Standards and Technology (NIST) spectral library. Thiophene showed a retention time of 3.7 min. Retention times for derivatives are given in Table S2. Quantification was performed by external calibration using known concentrations of thiophene. Runs without a transition metal compound or without acetylene were performed for comparison.

## 3. Results

**Figure 1:**
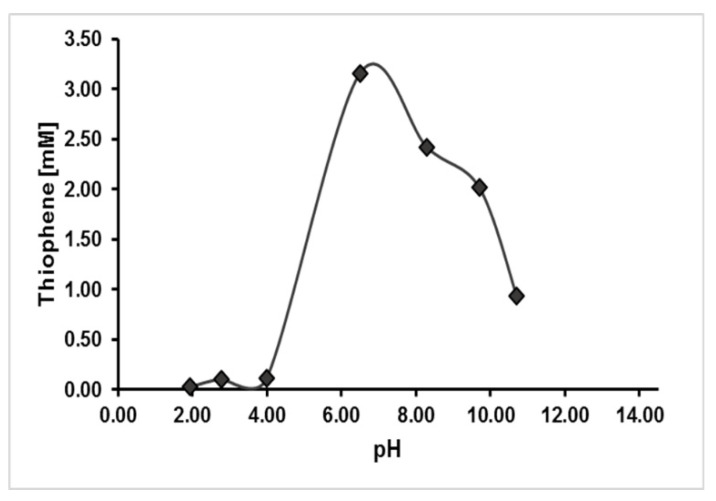
pH-Dependent formation of thiophene in the presence of NiS. Reactions were performed with 5.36 mmol acetylene and 1 mmol freshly precipitated nickel sulfide under aqueous conditions at 105 °C. Reactions were performed for 24 h and pH values were measured at the end of the reaction time.

**Figure 2:**
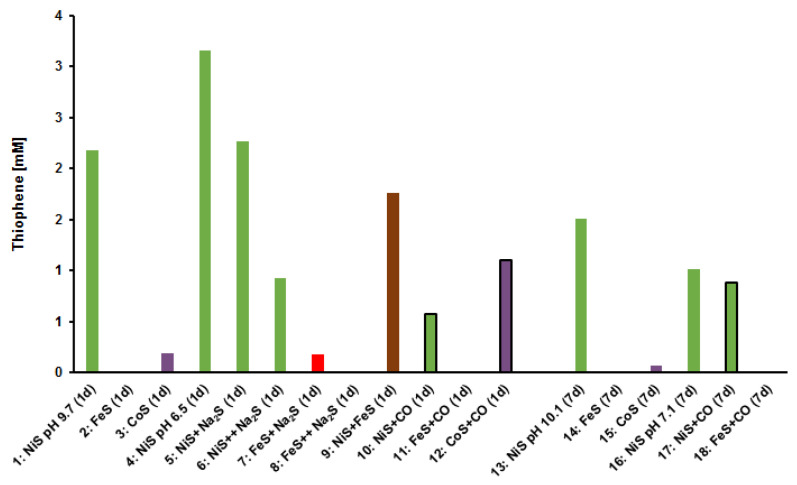
Total yield of thiophene formed in experiments as described in Table 1. Reactions were performed for 24 h (1 d) or 7 days (7 d). +Na_2_S/++Na_2_S^—^ imply free sulfide ions because of a higher molar ratio of Na_2_S to NiSO_4_. Bars are colored according to the metal sulfide used: NiS: green; FeS: red; CoS: purple; NiS/FeS: brown; Runs with CO show black frames.

We reacted acetylene at 105 °C for one day or seven days under strictly anoxic aqueous conditions, with freshly precipitated nickel sulfide, iron sulfide, cobalt sulfide, or mixtures thereof. In some runs, CO was added additionally as another putative reactant. The pH values were measured at the end of the reaction time (Table 1). After the indicated periods, the reaction mixtures were separated by centrifugation into a clear liquid supernatant and a black solid residue. Supernatants and solid residue were extracted separately using ethyl acetate. These extracts were finally analyzed by GC-MS. In a blank run without any addition of transition metal, under otherwise identical conditions to run 1, no formation of thiophene or thiophene derivatives was observed. Run 1, using NiS as the sulfide compound and catalyst at pH 9.7, is defined as standard run and was performed three times showing the formation of 2.2 mM thiophene as a mean concentration with a representative standard deviation of 14%. Under these conditions, thiophene formation was observed in a broad pH range from pH 5 to pH 11. However, yields were pH dependent as shown in Figure 1 and Table S1, with a pH optimum in the neutral range. Up to 3 mM thiophene were detected at pH 6.5 (run 4). Thiophene was also formed in comparable amounts in runs 3 and 10, using CoS or a mixed FeS/NiS catalyst, whereas only low amounts of thiophene were formed in the presence of FeS alone (Table 1, runs 2,7,8, and 14; Figure 2). Interestingly, in one third of the reactions, the amount of thiophene in the residue was up to two times higher than in the corresponding supernatants (Table 1) which reflects a strong binding of thiophene to the metal sulfide surfaces. This led us to the question as to whether the sulfur in thiophene derives from the solid NiS or, alternatively, from free sulfide in the solution. We therefore increased the amount of Na_2_S in runs 5–8. In run 5 which contained 0.5 mmol additional free sulfide, the amount of thiophene was not significantly changed (Table 1, run 5 vs. run 1). In run 6 with 1 mmol additional sulfide, the yield was diminished to one half (run 6 vs. run 1). This could again indicate that solid nickel sulfide served as the reacting agent and not the free sulfide, with a possible blockage of catalytic sites through excess sulfide. Otherwise, free sulfide ions shifted the pH to a more alkaline value which is less suited for thiophene formation (Figure 1). In the presence of FeS alone, only traces of thiophene were detected. In the presence of 0.5 mmol additional sulfide (run 7 vs. run 2), the thiophene yield was significantly enhanced, but again lowered in the presence of 1 mmol free sulfide (run 7 vs. 8). This could indicate a different reaction mechanism of FeS catalysis involving free sulfide ions and, possibly, a different pH dependency compared to NiS catalysis. Next, we chose a longer reaction time of 7 days, in an attempt to estimate reaction kinetics. We observed that the elongation of the reaction time in the NiS/acetylene system from 24h to one week was not favorable for thiophene formation (run 1 vs. 13, 3 vs. 15, 4 vs. 16), whereas thiophene yields increased in the NiS/acetylene/CO system (run 10 vs. 13) and the FeS/acetylene system (run 2 vs. 14). This showed that the formation of thiophene is a complex process influenced by many parameters, involving also consecutive reactions to other products. In Table 1, yields in mol% conversion based on acetylene are given additionally to the measured concentrations. The maximum conversion rate of 0.59% is reached for the NiS experiment at a nearly neutral pH value (run 4). For safety reasons the reactions were carried out at low pressure (1 bar). At a high sub-seafloor pressure (maybe >1000 bar), yields would be increased because of negative volumes of reaction [19,20].

**Figure 3:**
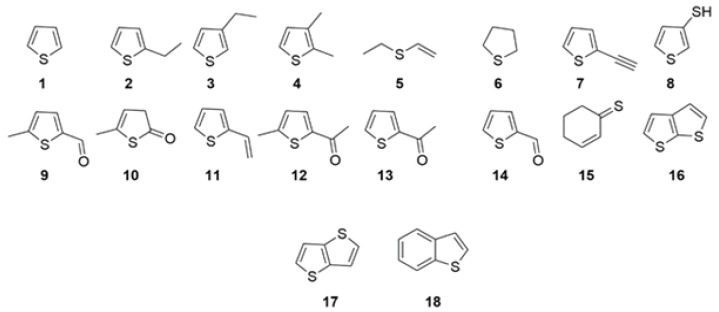
Thiophene and thiophene derivatives as formed from acetylene and nickel sulfide. Structures were verified by analytical standards and/or spectral libraries.

Further analysis by GC-MS led to the detection of several thiophene derivatives. Next to thiophene (1, Figure 3), 2-ethylthiophene (2), 3-ethylthiophene (3), 2,3-dimethylthiophene (4), ethyl-vinyl-sulfide (5), tetrahydrothiophene (6), 3-ethynylthiophene (7), 3-thiophenthiol (8), 5-methylthiophen-2-carboxaldehyde (9), 2[5H]-5-methylthiophenon (10), 2-vinylthiophene (11), 2-acetyl-5 methyl-thiophene (12), 2-acetylthiophene (13), thiophen-2-carboxaldehyde (14), cyclohex-2-enthion (15), cis-1,4-dithiapentalene (16), trans-1,4-dithiapentalene (17) and benzo[b]thiophene (18) were observed in the extracts of the reaction mixtures at estimated concentrations of 0.01–0.03 mM (Figure 3, Table S2). Product identification was performed by comparison with commercially available standards and/or comparison to the NIST14 database (Figure S1 and Figure S2, in the supplementary materials). Individual amounts of each derivative were not calculated, but ratios of thiophene to the total amount of thiophene derivatives are given in Table S2. Reactions performed in the presence of NiS showed ratios from 1.0–17.9, whereas the highest ratio of 25.4 was observed in the reaction setting using a mixed NiS/FeS catalyst. Reactions performed in the presence of CoS showed ratios in the range of 0.3 to 2.8, indicating higher amounts of derivatives in comparison to NiS. The low ratios in FeS settings (<0.8) were due to the low amounts of thiophene found in these settings. The decrease of the ratio thiophene to thiophene derivatives by addition of CO in the presence of NiS (run 1 vs. 10) could indicate follow up reactions initiated by CO. The fact, that no thiophene derivatives were detected after 7 days in the presence of CO could indicate reaction or degradation steps towards products, which are not covered by our experimental setup.

When thiophene was used as a starting material under conditions as described for run 11, tetrahydrothiophene (6), 2/3-ethylthiophene (1,2) and 5-methyl-thiophen-2-carboxaldehyde (9) were observed by GC-MS.

**Figure 4:**
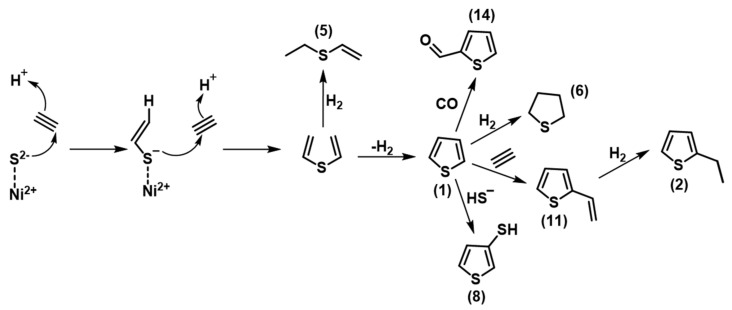
Hypothetical mechanism for the formation of thiophene and its derivatives from acetylene and nickel sulfide. ≡ signifies acetylene (C_2_H_2_)**.**

These findings demonstrate the formation of thiophene and its derivatives as products from acetylene and nickel sulfide under relatively mild hydrothermal conditions with the opportunity for further evolution. In Figure 4, a mechanism is proposed in analogy to the reaction of acetylene and hydrogen sulfide in super basic media [21]. In this scheme, the sulfur atom of NiS reacts with two molecules of acetylene in a concerted one-step mechanism. The so formed divinyl sulfide was not detected probably due to rapid conversion into by dehydrogenation to thiophene (1) or reduction to ethyl-vinyl-sulfide (5). Thiophene (1) could then react with further acetylene or sulfide to form 2-vinylthiophene (11), 2-ethylthiophene (2), 3-ethylthiophene (3) or 3-thiophenthiol (8). Further, it could be reduced to tetrahydrothiophene (6) or react with CO to form thiophen-2-carboxaldehyde (14). Additional experiments including stable isotope labelled precursors are required to unravel this mechanism in more detail. However, the various products observed under these conditions clearly imply that products downstream of thiophene are formed in a reaction network that could further evolve.

## 4. Discussion

Chemical reactivity on Earth and Mars could be determined by metal sulfide catalysis, e.g., by FeS catalysis, since both planets contain high amounts of iron in their mantles [22]. Nickel and cobalt as members of the iron group are often found together with iron and are also of special interest as catalysts in the “iron–sulfur theory” for the origin-of-life [23]. Earth’s core consists of 80–90% Fe-Ni alloys and 2.3wt% sulfur [24]. Fe-Ni sulfides are present on Earth as well as on Mars through ultramafic lava eruptions [25] and additional Ni is deposited on Mars through meteoritic impact [26]. On Earth, the formation of mixed NiFeS minerals, for example, of pentlandite (FeNi)_9_S_8_ and violarite FeNi_2_S_4_ is investigated in the context of serpentinization, a potential key process for metabolic evolution on early Earth [27].

In earlier work starting from acetylene, CO or cyanide under simulated hydrothermal conditions, we showed that nickel, especially NiS, is a potent catalyst for the formation of organic molecules, such as fatty acids, intermediates serving in biological carbon fixation and amino acids [11,12,28]. As we can now show, NiS catalyzes additionally the formation of thiophene from acetylene. The observed low thiophene formation with FeS underlines the importance of nickel minerals in this context.

Organic sulfur compounds in general are indeed essential for life and play an important role in the sulfur cycle on Earth [29]. In addition to thiophene, methanethiol and carbonyl sulfide can be found in terrestrial hydrothermal exhalations and were also used for simulated primordial synthesis of biomolecules [23,30]. In the biological context, thiophene and its derivatives (such as benzo-thiophenes) are sometimes considered as secondary biomarkers, preserving the original n-alkane chain or carbon skeleton of biomolecules in sulfur-bound forms at different lithofacies [31]. Organic sulfur compounds are more stable under sulfide rich geological conditions than their biological precursors (e.g., functionalized lipids), and can therefore be found in ancient sedimentary rocks [32].

In extant biochemistry, thiophene is still conserved as a structural part of biotin, which is a prosthetic group for carboxylase classed enzymes, like the pyruvate carboxylase [33]. Furthermore, thiophenes can be found as structural components in the quinone fractions (e.g., caldariellaquinone—benzo[b]thiophene-4,7-quinone) of extreme thermophilic and acidophilic archaeons, like *Caldariella acidiphila* and *Sulfolobus solfataricus* [34,35].

According to recent literature [7,36], thiophenes *per se* should not be named biomarkers, due to their possible abiotic origin, but the presence of thiophenes, as easily detectable molecules, could indicate samples or sites, which should be investigated in more detail for the presence of additional organic molecules like amino acids or fatty acids. It is also suggested that terrestrial origin-of-life conditions could be used as a guideline in the search for life on extraterrestrial planets [37].

## 5. Conclusions

We here could show the abiotic formation of thiophenes from acetylene and transition metal sulfides under aqueous conditions, which we consider as a valid simulation of volcanic hydrothermal settings on early Earth, but also on other planets. Under identical conditions, we could previously demonstrate the conversion of acetylene and CO into short chain fatty acids (C_3_–C_9_) and other C_2_–C_4_ compounds including metabolic intermediates of carbon fixation in extant life [9,10]. These compounds are also considered as important precursors for a potential chemo-autotrophic origin-of-life. It is therefore tempting to speculate that the detection of thiophenes on planets reflects possible habitats for the early emergence and evolution of metabolism and life.

## Figures and Tables

**Table 1:** Transition metal catalyzed formation of thiophene. Reactions were performed with 120 mL (5.36 mmol) or 60 mL (2.68 mmol) acetylene and freshly precipitated sulfides under aqueous conditions at 105 °C. NiSO_4_, CoSO_4_ and FeSO_4_ were used as hydrates (see method section). Reactions were performed for 24 h (run 1–12) or 7 days (run 13–18). pH-Values were measured at the end of the reaction time. Run 1 was performed three times showing a representative standard deviation of 14%. Thiophene concentrations were given in mM for the separated organic extracts of supernatants and solid sulfides as well as total concentration in the 5 mL setups. Yields are given in mol% conversion based on acetylene.

Run	NiSO_4_	FeSO_4_	CoSO_4_	Na_2_S	NaOH	CO	C_2_H_2_	pH_end_	Extract Supernatant	Extract Solid	Total Conc.	Total Yield
	(mmol)	(mmol)	(mmol)	(mmol)	(mmol)	(mL)	(mL)		(mM)	(mM)	(mM)	(%)
**1**	1			1	0.5	-	120	9.7	0.379	0.709	2.175	0.406
**2**	-	1	-	1	0.5	-	120	9.0	<0.001	<0.001	<0.001	<0.001
**3**	-	-	1	1	0.5	-	120	9.0	0.047	0.046	0.185	0.035
**4**	1	-	-	1	-	-	120	6.5	0.337	1.243	3.160	0.590
**5**	1	-	-	1.5	-	-	120	11.0	0.393	0.740	2.268	0.423
**6**	1	-	-	2	-	-	120	13.5	0.333	0.129	0.925	0.173
**7**	-	1	-	1.5	-	-	120	12.0	0.023	0.066	0.177	0.033
**8**	-	1	-	2	-	-	120	13.5	0.002	0.002	0.008	0.001
**9**	0.5	0.5	-	1	0.5	-	120	11.0	0.377	0.503	1.761	0.329
**10**	1	-	-	1	0.5	60	60	9.5	0.220	0.067	0.574	0.214
**11**	-	1	-	1	0.5	60	60	9.0	0.002	<0.001	0.004	0.001
**12**	-	-	1	1	0.5	60	60	9.5	0.075	0.476	1.103	0.412
**13**	1	-	-	1	0.5	-	120	10.1	0.135	0.618	1.505	0.281
**14**	-	1	-	1	0.5	-	120	8.5	0.001	0.001	0.003	0.001
**15**	-	-	1	1	0.5	-	120	8.7	0.023	0.012	0.070	0.013
**16**	1	-	-	1	-	-	120	7.1	0.040	0.468	1.015	0.190
**17**	1	-	-	1	0.5	60	60	7.8	0.225	0.213	0.877	0.327
**18**	-	1	-	1	0.5	60	60	7.6	0.000	0.001	0.002	0.001

## Supplementary Materials

The following are available online at https://www.mdpi.com/2075-1729/11/2/149/s1, Table S1: pH dependent formation of thiophene in the presence of NiS. Table S2: Identified thiophene derivatives and their retention times. Figure S1: GC/MS chromatograms comparing thiophene and commercially available thiophene derivatives, Figure S2: GC/MS mass spectra comparing reaction products to commercially available thiophene standards and mass spectra from NIST14 library.
