# Supplementary material for: Formation of Thiophene under Simulated Volcanic Hydrothermal Conditions on Earth—Implications for Early Life on Extraterrestrial Planets?
Source: Life (Basel). 2021 Feb 16;11(2):149. doi: 10.3390/life11020149 (PMC7920246; doi:10.3390/life11020149)
Supplement: Supplementary file 1 [file life-11-00149-s001.pdf]

# Supplemental Materials

**Table S1:** pH dependent formation of thiophene in the presence of NiS.

**Table S2:** Identified thiophene derivatives and their retention times.

**Figure S1:** GC/MS chromatograms comparing thiophene and commercially available thiophene derivatives.

**Figure S2:** GC/MS mass spectra comparing reaction products to commercially available thiophene standards and mass spectra from NIST14 library.

**Table S1. pH dependent formation of thiophene in the presence of NiS**. Reactions were performed with 5.36 mmol acetylene and 1 mmol freshly precipitated nickel sulfide under aqueous conditions at 105°C. Reactions were performed for 1d and pH values were measured at the end of the reaction time.

| pH | $H_2SO_4$ [mmol] | NaOH [mmol] | Supernatant [mM] | Solid [mM] | Total [mM] |
|---|---|---|---|---|---|
| 1.9 | 1.0 | - | 0.001 | 0.013 | **0.03** |
| 2.8 | 0.5 | - | 0.027 | 0.025 | **0.11** |
| 4.0 | 0.2 | - | 0.003 | 0.051 | **0.11** |
| 6.5 | - | - | 0.336 | 1.242 | **3.16** |
| 8.3 | - | 0.5 | 0.060 | 1.148 | **2.42** |
| 9.7 | - | 1.0 | 0.091 | 0.971 | **2.02** |
| 10.7 | - | 1.5 | 0.212 | 0.255 | **0.94** |

**Table S2: Identified thiophene derivatives (x) and their retention times (RT, min)** from experiments with acetylene and metal sulfides as described in Table 1. Numbers corresponding to Figure 3 are given in brackets. The amounts were not quantified, but indicated by x, if detected. The ratio thiophene to thiophene derivatives is calculated from peak areas.

| **Run** | **RT [min]** | **1** | **2** | **3** | **4** | **5** | **6** | **7** | **8** | **9** | **10** | **11** | **12** | **13** | **14** | **15** | **16** | **17** | **18** |
|---|---|---|---|---|---|---|---|---|---|---|---|---|---|---|---|---|---|---|---|
| **Product** | | | | | | | | | | | | | | | | | | | |
| **2-Ethylthiophene (2)** | 7.5 | x | x | x | x | x | x | x | x | x | x | x | x | x | - | - | x | - | - |
| **3-Ethylthiophene (3)** | 8.2 | x | - | x | x | x | x | - | - | x | x | - | x | - | - | - | - | - | - |
| **2,3-Dimethylthiophene (4)** | 7.7 | x | | x | x | x | x | - | - | x | x | - | x | | | | | | |
| **Ethylvinylsulfide (5)** | 4.0 | - | - | - | - | | - | - | | - | - | - | - | - | - | - | - | - | - |
| **Tetrahydrothiophene (6)** | 6.2 | x | x | x | - | x | x | x | x | - | x | x | x | - | - | - | - | - | - |
| **3-Ethynylthiophene (7)** | 8.0 | - | - | - | - | | - | - | - | - | - | - | - | - | - | - | - | - | - |
| **3-Thiophene thiol (8)** | 10.2 | x | x | x | x | x | x | x | - | x | x | - | x | x | - | x | x | - | - |
| **5-Methylthiophene-carboxaldehyde (9)** | 11.5 | - | - | x | - | | - | - | - | - | x | - | - | x | x | x | - | - | - |
| **2[5H]-5-Methylthiophenon (10)** | 11.8 | - | - | - | - | | - | - | - | - | x | - | x | - | - | - | - | - | - |
| **2-Vinylthiophene (11)** | 8.1 | x | - | x | x | x | x | - | - | x | x | - | x | x | - | - | x | - | - |
| **2-Acetyl-5-methylthiophene (12)** | 12.5 | - | - | - | - | | - | - | - | - | - | - | x | - | - | - | - | - | - |
| **2-Acetylthiophene (13)** | 11.5 | x | - | - | x | x | - | - | - | - | - | - | x | - | - | - | - | - | - |
| **Thiophene-carboxaldehyde (14)** | 9.9 | - | - | - | - | | - | - | - | - | x | - | - | - | - | - | - | - | - |
| **Cyclohex-2-enthion (15)** | 9.2 | x | - | x | x | x | x | - | - | x | x | - | x | - | - | - | - | - | - |
| **cis-1,4-Dithiapentalene (16)** | 13.5 | x | - | - | x | x | x | x | x | x | x | - | x | - | - | - | - | - | - |
| **trans-1,4-Dithiapentalene (17)** | 13.6 | x | - | x | x | x | x | - | - | x | x | - | x | - | - | - | - | - | - |
| **Benzo[*b*]thiophene (18)** | 13.3 | x | - | x | x | x | x | - | - | x | x | - | x | - | - | - | - | - | - |
| **Ratio thiophene : Σthiophene derivatives** | | **12.5** | **<0.1** | **0.3** | **9.6** | **19.3** | **2.7** | **<0.1** | **0.8** | **25.4** | **1.3** | **0.1** | **3.2** | **17.9** | **0.1** | **2.8** | **1.0** | **-** | **-** |

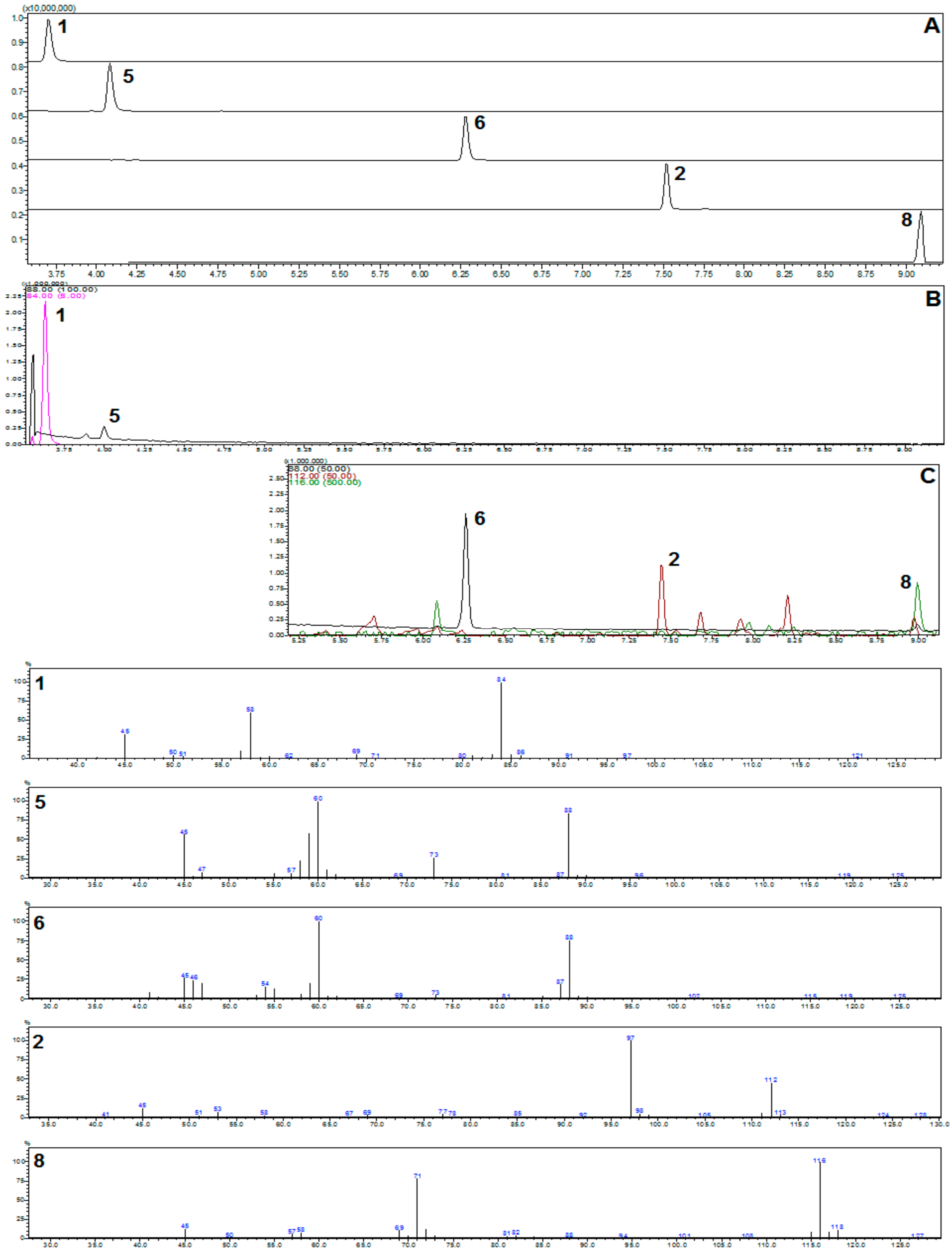

**Figure S1: GC/MS chromatograms comparing thiophene and commercially available thiophene derivatives** (**A**) to reaction products (**B,C**). Numbers correspond to structures in Figure 3

**Thiophene (1)          similarity to NIST14 database: 96%**

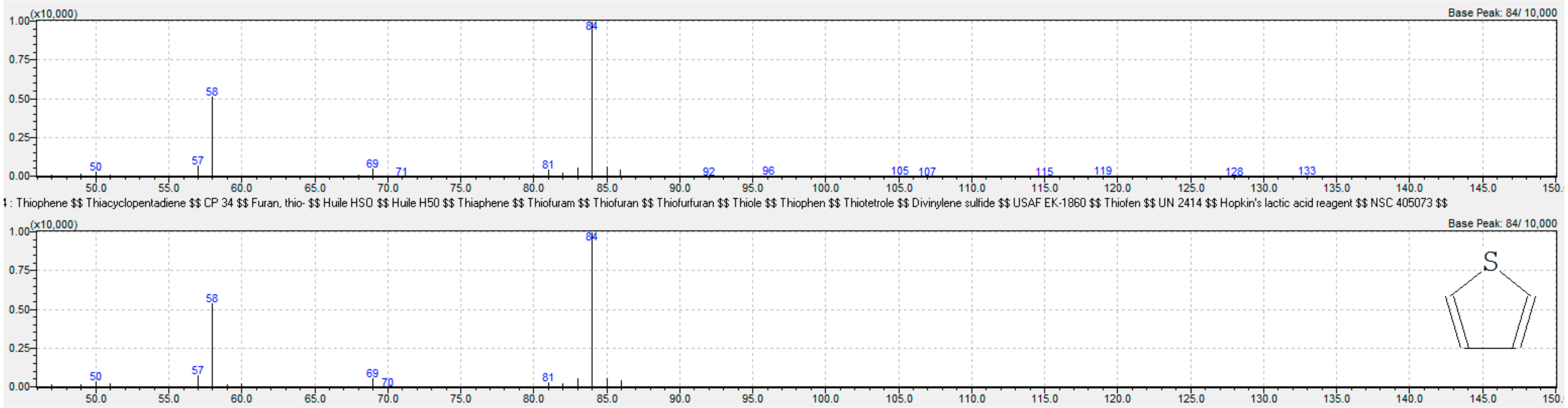


**Thiophene analytical standard (Sigma Aldrich)**

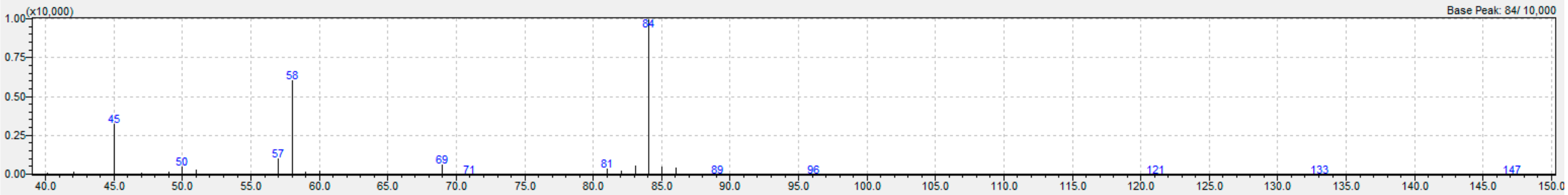

**2-Ethylthiophene (2)** **similarity to NIST14 database: 93%**

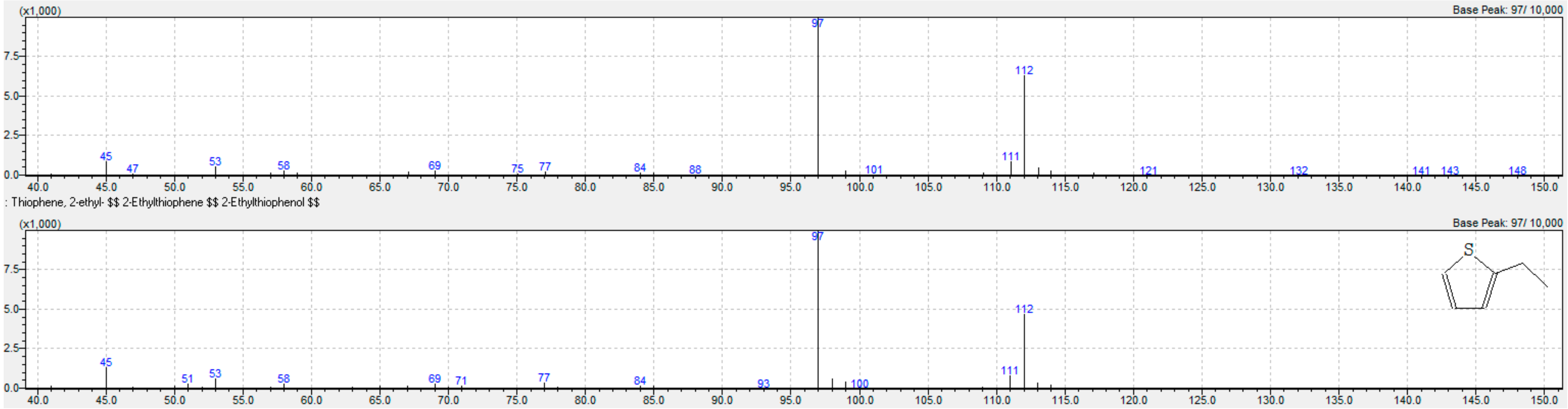


**3-Ethylthiophene (3)** **similarity to NIST14 database: 91%**

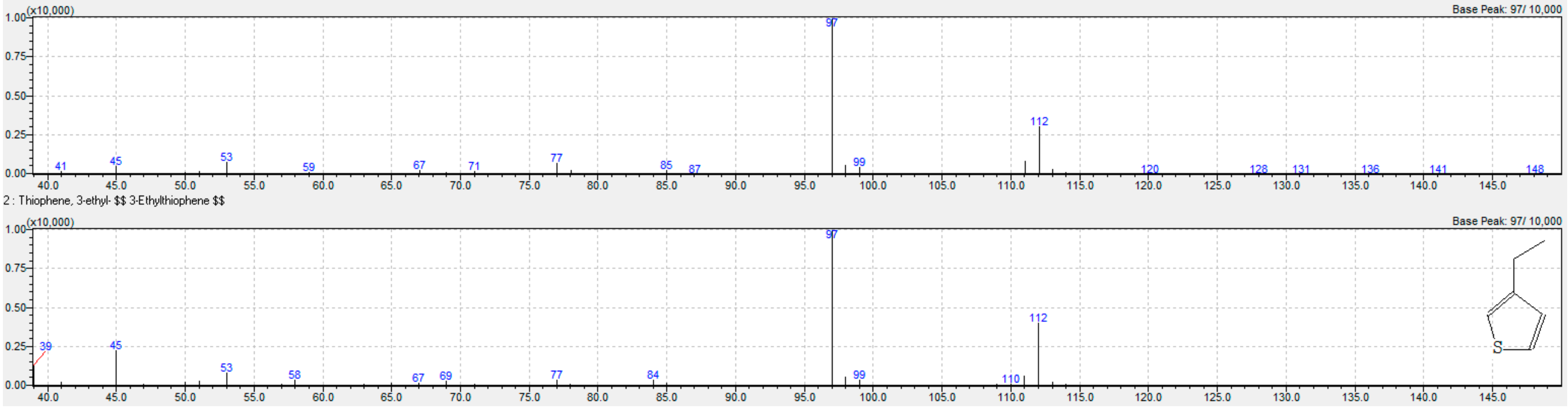

**2,3-Dimethylthiophene (4)   similarity to NIST14 database: 82%**

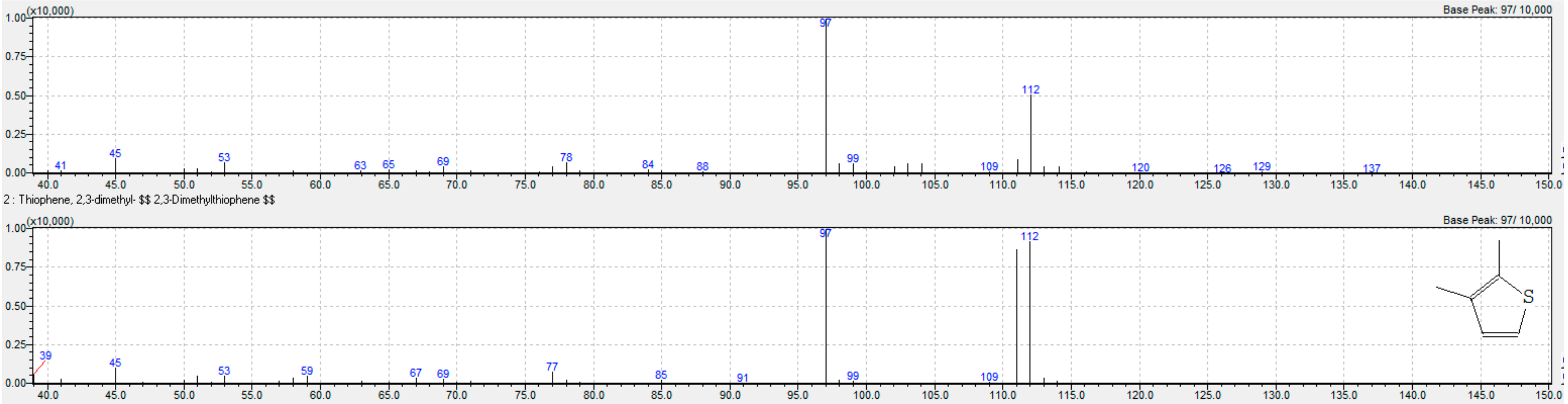

**Tetrahydrothiophene (6)     similarity to NIST14 database: 92%**

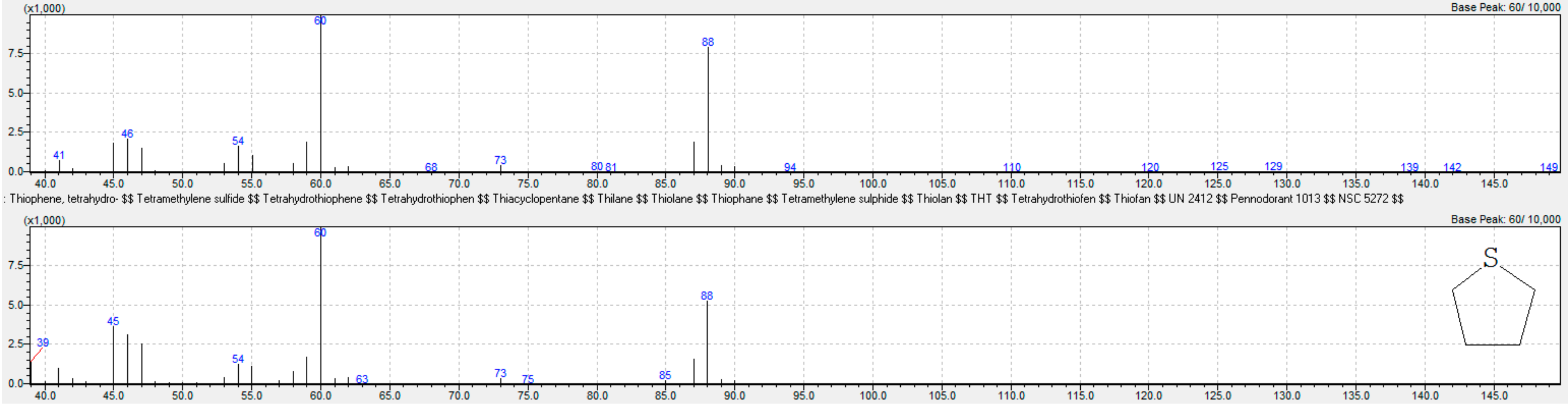


**Tetrahydrothiophene analytical standard (Sigma Aldrich)**

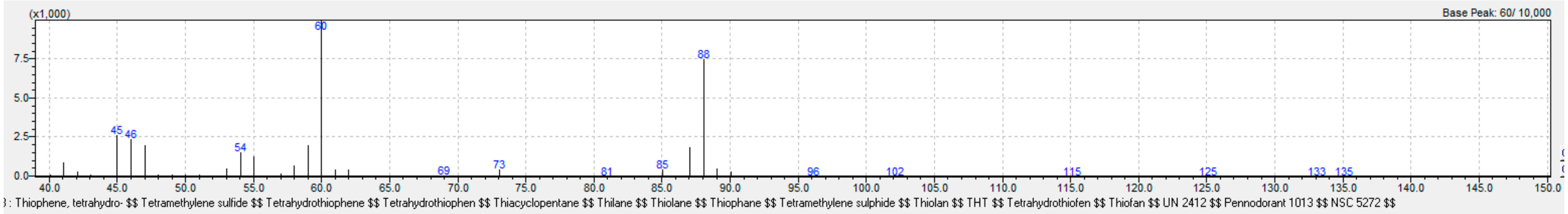

## 2-Thiophenthiol (8)  similarity to NIST14 database: 93%

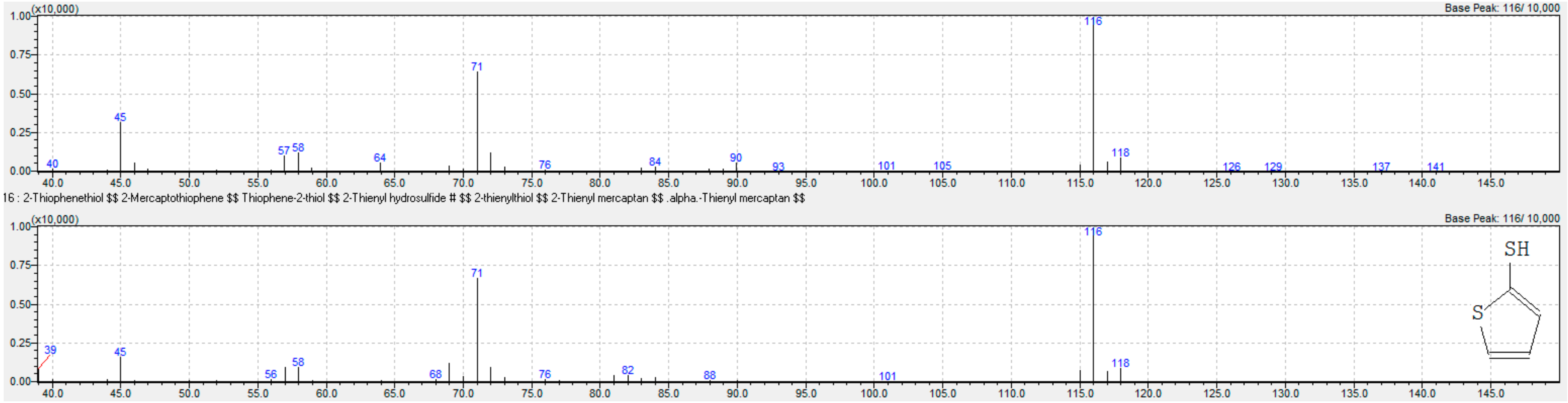


## 2-Thiophenthiol analytical standard (Sigma Aldrich)

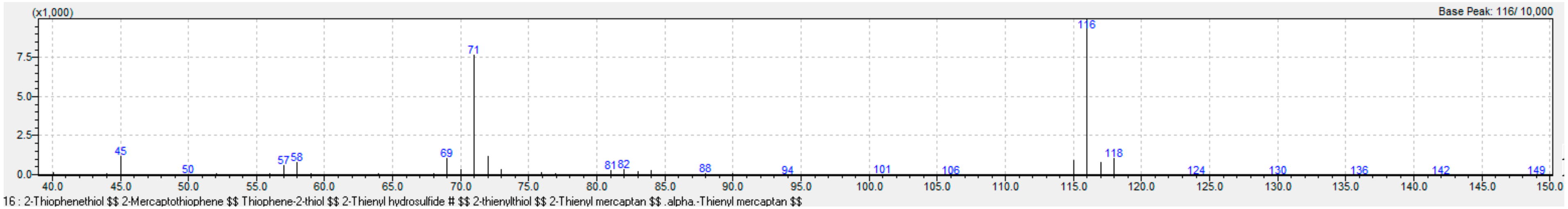

**5-Methylthiophene-carboxaldehyde (9)    similarity to NIST14 database: 82%**

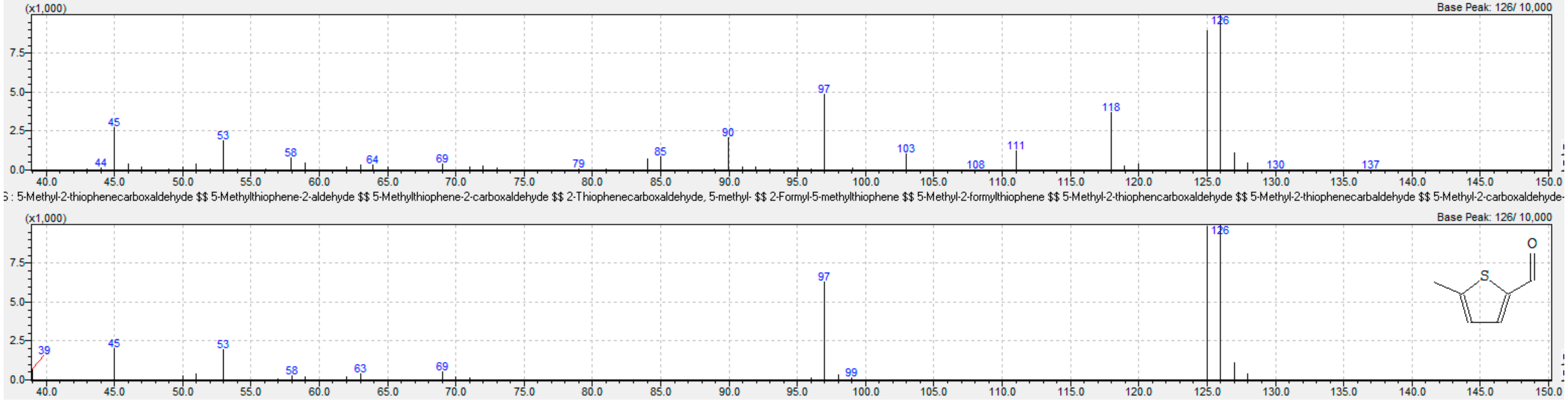


**2[5H]-5-Methylthiophenon (10)    similarity to NIST14 database: 77%**

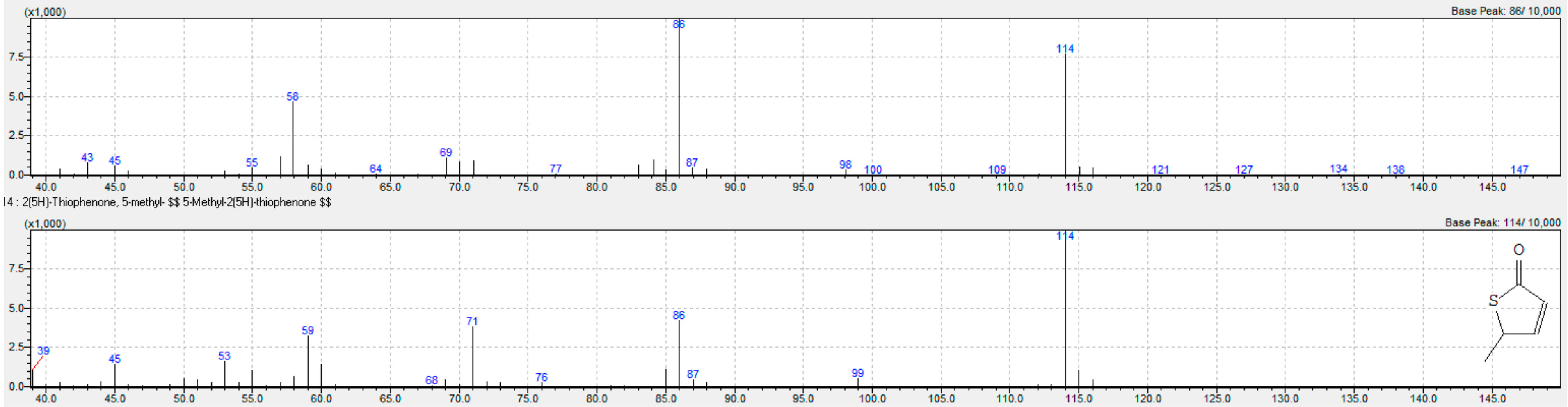

**2-Vinylthiophene (11) similarity to NIST14 database: 81%**

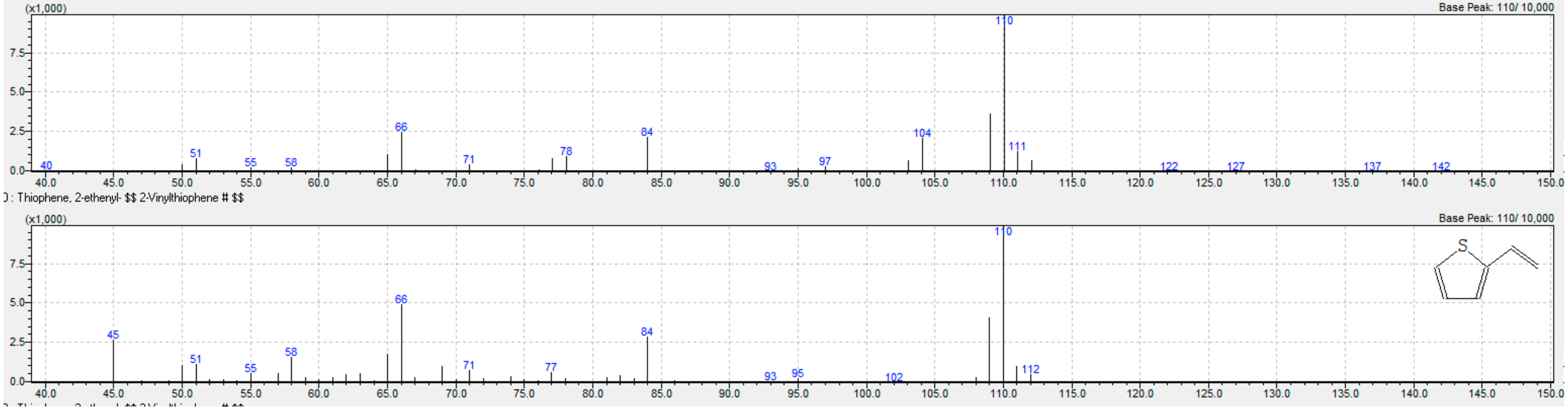


**2-Acetyl-5-methylthiophene (12) similarity to NIST14 database: 93%**

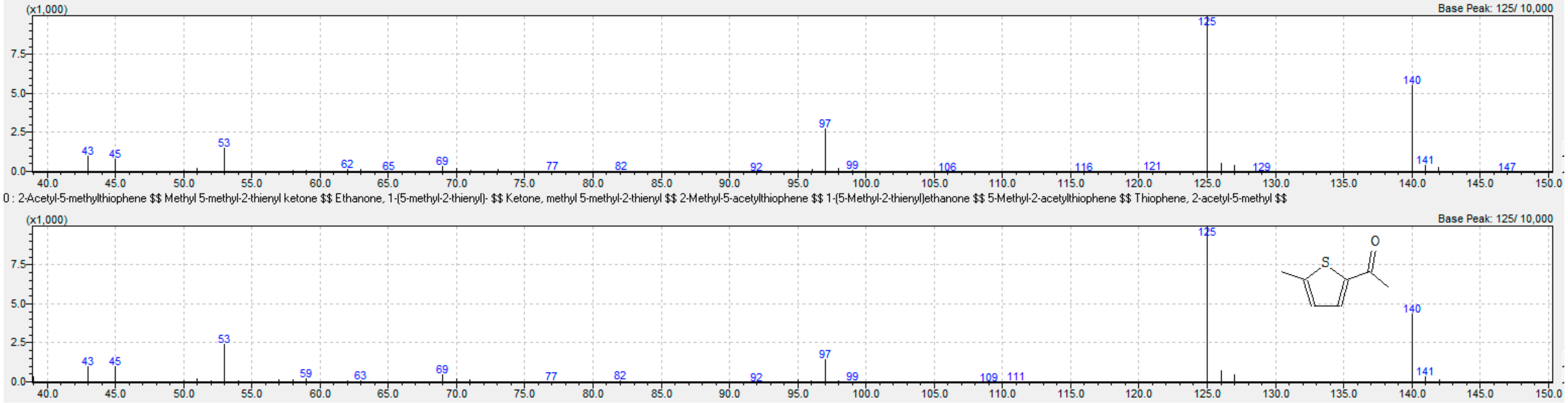

**2-Acetylthiophene (13)  similarity to NIST14 database: 78%**

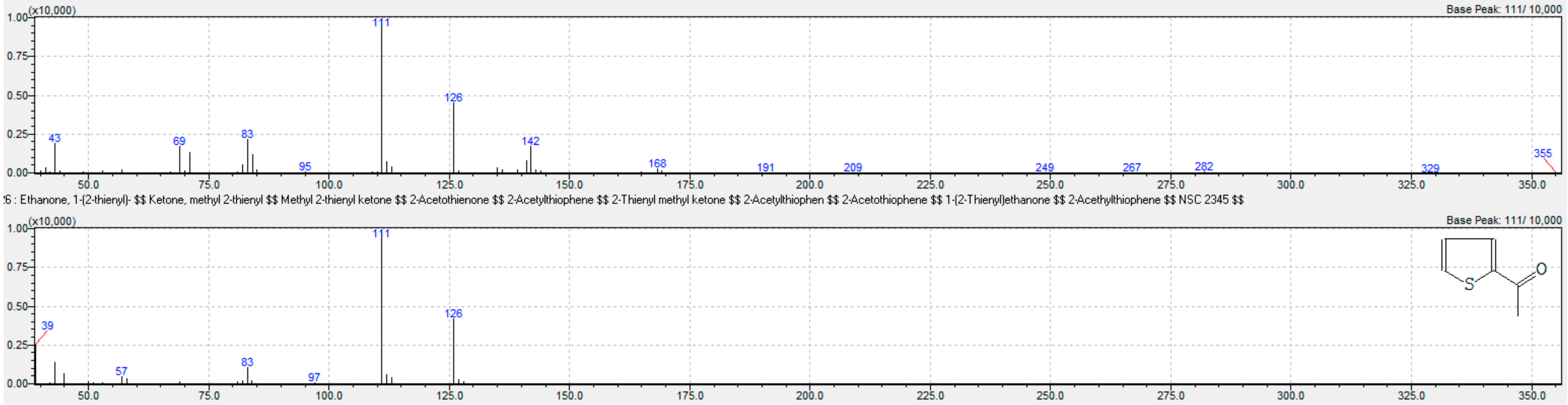

**Thiophene-2-carboxaldehyde (14) similarity to NIST14 database: 84%**

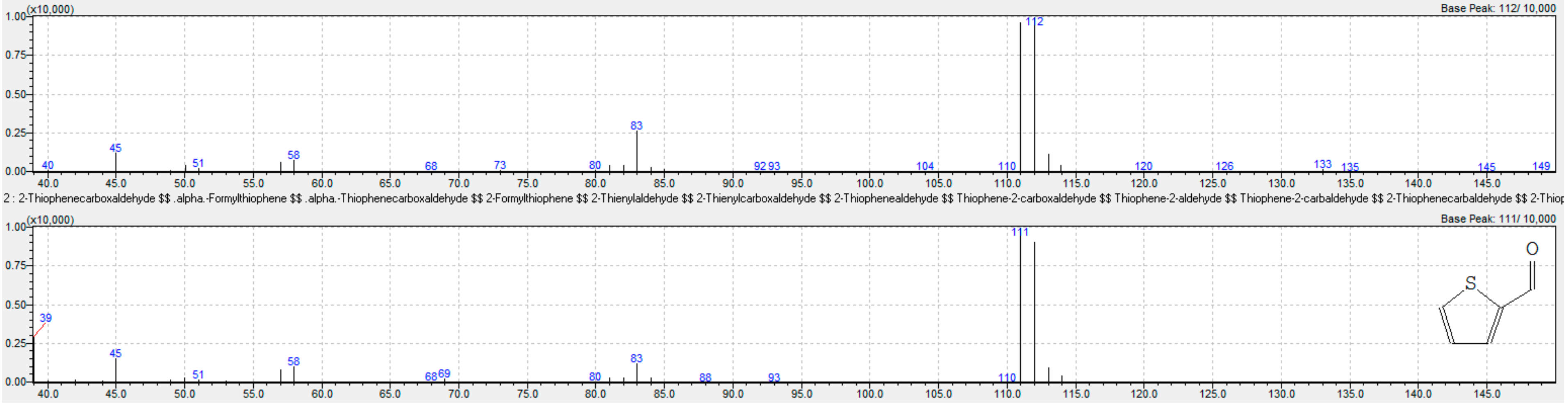


**Thiophene-2-carboxaldehyde analytical standard (Sigma Aldrich)**

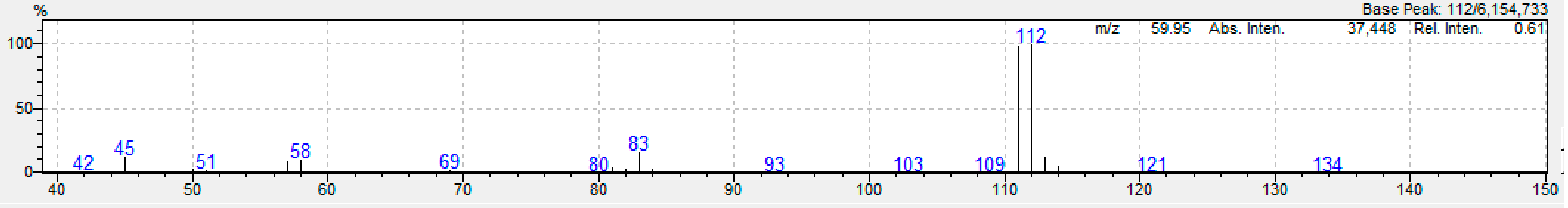

**Cyclohex-2-enthion (15)** **similarity to NIST14 database: 78%**

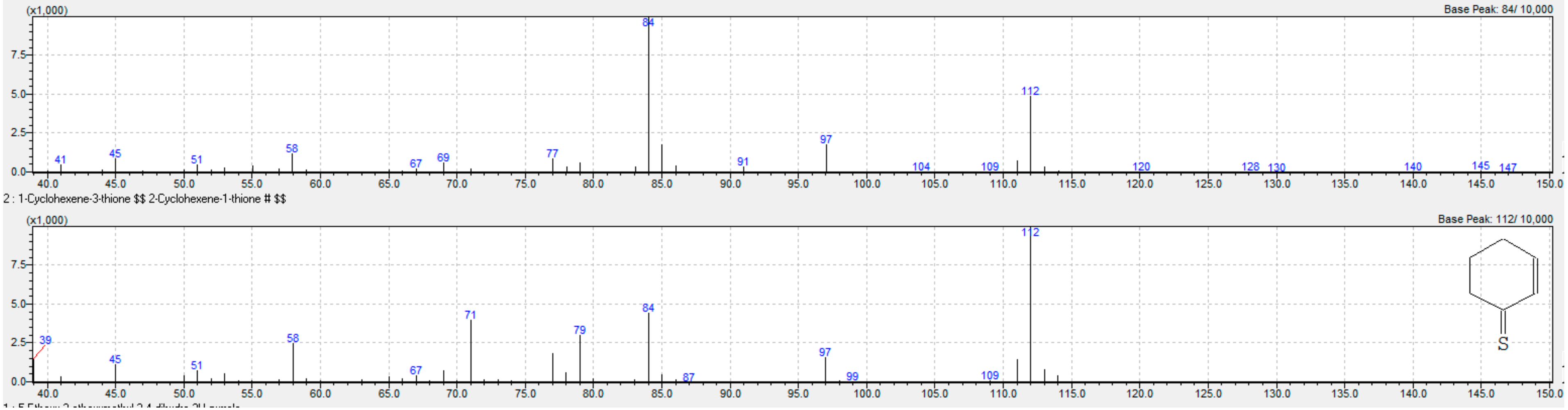


**cis-1,4-Dithiapentalene (16)** **similarity to NIST14 database: 89%**

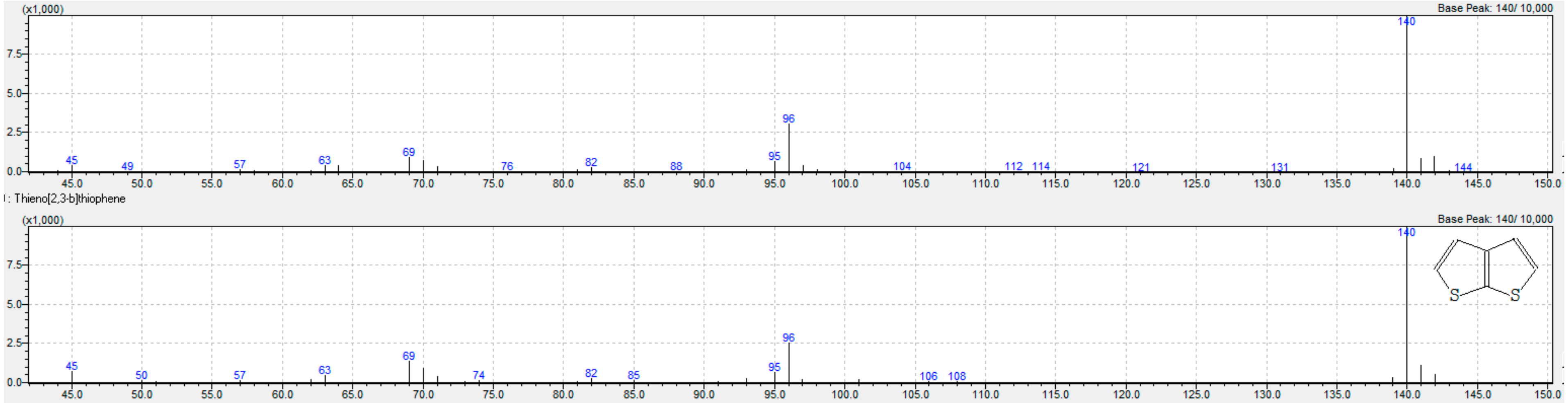

**trans-1,4-Dithiapentalene (17)** **similarity to NIST14 database: 92%**

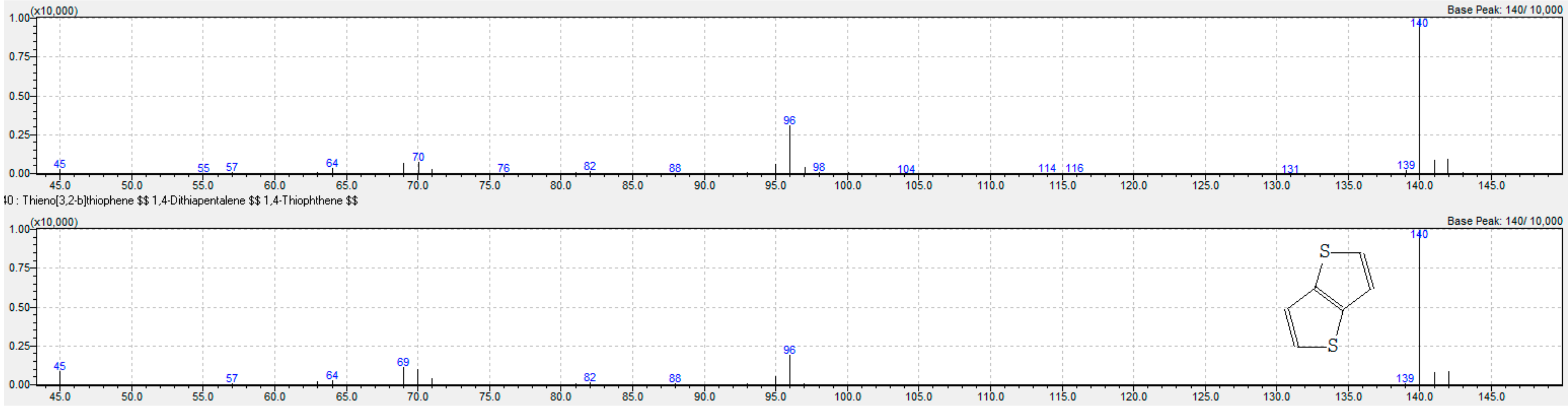

**Benzo[*b*]thiophene (18)** **similarity to NIST14 database: 97%**

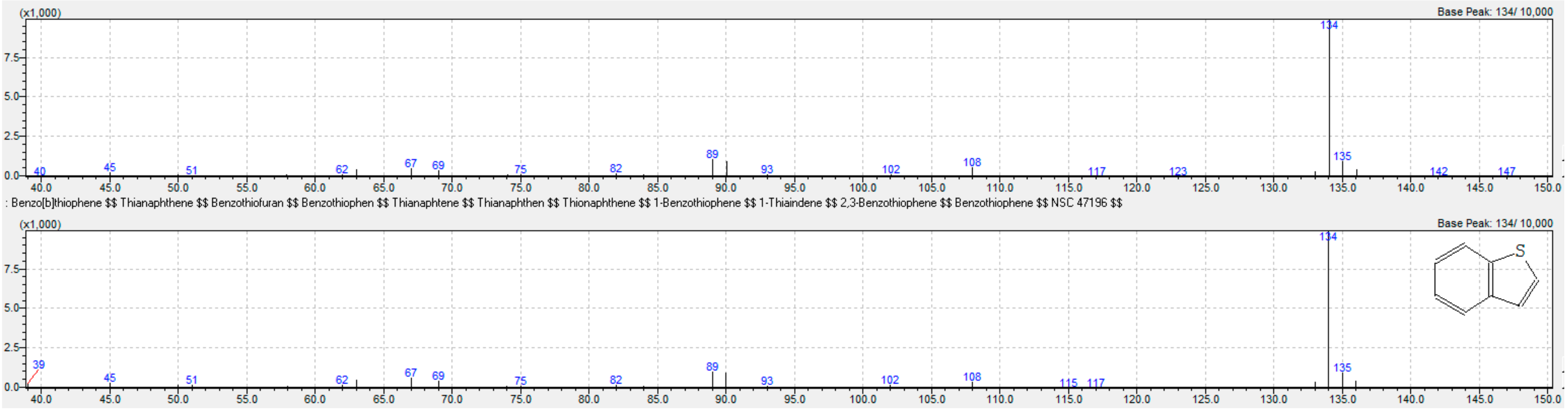


**Figure S2: GC/MS mass spectra comparing reaction products to commercially available thiophene standards and mass spectra from NIST14 library**. Numbers corresponding to Figure 3 in brackets.
